# Reduced basal and increased topdressing fertilizer rate combined with straw incorporation improves rice yield stability and soil organic carbon sequestration in a rice–wheat system

**DOI:** 10.3389/fpls.2022.964957

**Published:** 2022-08-26

**Authors:** Jianwei Zhang, Jidong Wang, Yan Zhou, Lei Xu, Yinglong Chen, Yanfeng Ding, Yunwang Ning, Dong Liang, Yongchun Zhang, Ganghua Li

**Affiliations:** ^1^Scientific Observing and Experimental Station of Arable Land, Ministry of Agriculture and Rural/National Agricultural Experimental Station for Agricultural Environment/Institute of Agricultural Resources and Environment, Jiangsu Academy of Agricultural Sciences, Nanjing, China; ^2^National Engineering and Technology Center for Information Agriculture/Key Laboratory of Crop Physiology and Ecology in Southern China/Jiangsu Collaborative Innovation Center for Modern Crop Production, Nanjing Agricultural University, Nanjing, Jiangsu, China; ^3^School of Agricultural Equipment Engineering, Jiangsu University, Zhenjiang, China; ^4^The UWA Institute of Agriculture and School of Agriculture and Environment, The University of Western Australia, Perth, WA, Australia

**Keywords:** long–term experiment, fertilizer management, grain yield stability, soil organic carbon sequestration, rice–wheat system

## Abstract

Fertilizer management is vital for sustainable agriculture under climate change. Reduced basal and increased topdressing fertilizer rate (RBIT) has been reported to improve the yield of in–season rice or wheat. However, the effect of RBIT on rice and wheat yield stability and soil organic carbon (SOC) sequestration potential is unknown, especially when combined with straw incorporation. Here, we report the effect of RBIT with/without straw incorporation on crop yields, yield stability, SOC stock, and SOC fractions in the lower Yangtze River rice–wheat system region over nine years. RBIT with/without straw incorporation significantly increased nine–year average and annual rice yields but not wheat yields. Compared with conventional fertilization (*CF*), RBIT did not significantly affect wheat or rice yield stability, but combined with straw incorporation, it increased the sustainable yield index (SYI) of wheat and rice by 7.6 and 12.8%, respectively. RBIT produced a higher C sequestration rate (0.20 Mg C ha^−1^ year^−1^) than *CF* (0.06 Mg ha^−1^ year^−1^) in the 0–20 cm layer due to higher root C input and lower C mineralization rate, and RBIT in combination with straw incorporation produced the highest C sequestration rate (0.47 Mg ha^−1^ year^−1^). Long–term RBIT had a greater positive effect on silt+clay (0.053 mm)–associated C, microbial biomass C (MBC), dissolved organic C, and hot water organic C in the surface layer (0–10 cm) than in the subsurface layer (10–20 cm). In particular, the increases in SOC pools and mean weight diameter (MWD) of soil aggregates were greater when RBIT was combined with straw incorporation. Correlation analysis indicated that topsoil SOC fractions and MWD were positively correlated with the SYI of wheat and rice. Our findings suggest that the long–term application of RBIT combined with straw incorporation contributed to improving the sustainability of rice production and SOC sequestration in a rice–wheat system.

## Introduction

The rice–wheat system is the most important cropping system in southeastern Asia, with an area of 24 million hectares (Ladha et al., 2003). However, according to Ladha et al. (2003), rice and wheat yields have stagnated in 72 and 85% of Asia’s rice–wheat systems. Ray et al. (2015) reported that year–to–year climate variability explained ~32% and ~36% of rice and wheat yield variability globally, respectively (Ray et al., 2015), suggesting that inter–annual climate variations greatly affect crop yield stability (Wang et al., 2017; Fatima et al., 2022). Some experiments indicated that poor management reduced the sustainable production of crops due to soil degradation (i.g., soil organic carbon (SOC) decline, soil compaction, and soil acidification) (Ladha et al., 2003; Zhang et al., 2015; Rubio et al., 2021). Moreover, data from 49 field experiments revealed that surface soil horizons would lose 30–203 petagrams of C under a 1°C warming scenario (Crowther et al., 2016), implying that climate change increases the risk of SOC loss. Therefore, optimal agricultural management improving crop yield stability and SOC content in the rice–wheat system will be very important under climate change (Ladha et al., 2003; Han et al., 2020; Bhatt et al., 2021; Fatima et al., 2022).

Fertilization management plays a vital role in the rice–wheat system (Ladha et al., 2003; Han et al., 2020; Bhatt et al., 2021). Reduced basal and increased topdressing fertilizer rate (RBIT) is an effective way and has been recommended in some rice and wheat planting countries, including China (Shi et al., 2012; Sui et al., 2013; Chen et al., 2015; Wang et al., 2019; Zhang et al., 2021; Ye et al., 2022), Japan (Myint et al., 2009; Kamiji et al., 2011; Moe et al., 2014), India (Khurana et al., 2008; Jat et al., 2012), Australia (Wood et al., 2020), Italy (Ercoli et al., 2013), Spain (Garrido-Lestache et al., 2005) and Argentina (Melaj et al., 2003). Short-term (2–3 years) field experiments showed that RBIT could generally increase in-season rice or wheat yield (Melaj et al., 2003; Kamiji et al., 2011; Jat et al., 2012; Shi et al., 2012; Ercoli et al., 2013; Chen et al., 2015; Wang et al., 2019; Wood et al., 2020; Zhang et al., 2021; Ye et al., 2022). This due to that RBIT provided better synchronization of rice and wheat nitrogen (N) demand with N supply, improving crop N uptake and promoting spikelet differentiation and carbohydrate synthesis, compared with conventional fertilization (*CF*) (Shi et al., 2012; Ercoli et al., 2013; Sui et al., 2013; Xu et al., 2015; Zhang et al., 2021). However, some studies found that N fertilizer timing and splitting had no effect on wheat yields, due to excessive rainfall and low temperature in the autumn-winter period and impeding the establishment and tillering of crop (Garrido-Lestache et al., 2005; Pampana and Mariotti, 2021). High N concentration in plant under frequent cloudy and rain may increase the risk of lodging and late maturity, leading to yield loss, implying that RBIT may increase the fluctuation of year–to–year crop yield under climate change. Especially the intensity and frequency of extreme temperature and precipitation are expected to increase due to climate change (IPCC, 2014, 2021). Therefore, it is necessary to effectively evaluate the effect of RBIT on crop yield stability in the rice–wheat system.

Long–term experiments offer important advantages relative to short–term studies in providing valuable information on the effect of fertilization management on the sustainability of crop production (Manna et al., 2005; Qaswar et al., 2019; Han et al., 2020). Studies have reported that the sustainable yield index (SYI) of rice and wheat has a positive relationship with SOC content (Wanjari et al., 2004; Qaswar et al., 2019). These findings suggest that SOC plays an important role in improving crop yield stability under climate change (Manna et al., 2005; Han et al., 2020; Shi et al., 2022). However, ^15^N tracer tests found that the residual soil N derived from basal fertilizer was higher in the paddy (Wang et al., 2018) and upland soil (Shi et al., 2012) at harvest than from topdressing fertilizer. RBIT with low residual soil N from fertilizer N and high crop nutrient removal (Khurana et al., 2008; Sui et al., 2013; Xu et al., 2015) may cause a decrease in soil nutrient content, which in turn stimulates the mineralization of soil organic matter and releases more soil available nutrients for crop growth. Thus, we speculate that the continuous application of this practice may reduce SOC content, which in turn is not conducive to the sustainability of crop production in the rice–wheat system.

Straw incorporation is a common practice to improve the sustainability of crop production in the rice–wheat system (Sharma and Prasad, 2008; Huang et al., 2013; Zhu et al., 2015; Li et al., 2018; Han et al., 2020; Jin et al., 2020; Mohamed et al., 2021). The global meta-analysis showed that straw incorporation increased the SOC by 12.0%, easily oxidizable C (EOC) by 24.4%, microbial biomass C (MBC) by 26.7%, and dissolved organic C (DOC) by 24.2% (Liu et al., 2014). Some studies showed that straw incorporation could increase and stabilize crop yield in wheat–maize rotation (Shi et al., 2022) and rice-bases cropping system (Qaswar et al., 2019) due to improving SOC. Some studies indicated that under straw incorporation conditions, N fertilizer rate should be increased in the early stage of growth to mitigate microbial competition for N with crops (Cao et al., 2018; Li et al., 2018; Wei et al., 2019; Zheng et al., 2019). However, there is a lack of effective information on whether straw incorporation with RBIT impacts the sustainability of crop production in rice–wheat systems. We therefore performed a 9–year field experiment to assess the effect of continuous application of RBIT with or without straw incorporation on crop yield, yield stability, and SOC sequestration potential in rice-wheat systems.

## Materials and methods

### Experiment site

The field experiment was conducted at the Danyang Experimental Station in Zhenjiang, Jiangsu province, eastern China (119°10′, 34°36′). This area has a subtropical humid monsoon climate with an average air temperature of 14–15°C and annual precipitation of 800–1,100 mm. The soil type was classified as waterloggogenic paddy soil (Chinese Soil Taxonomy). Before starting the experiment in 2009, the main properties of the soil (0–20 cm) were: SOC, 10.07 g kg^−1^; total N, 0.97 g kg^−1^; NH_4_OAc K, 93.50 mg kg^−1^; and Olsen P, 13.60 mg kg^−1^.

### Experimental design

**Figure 1:**
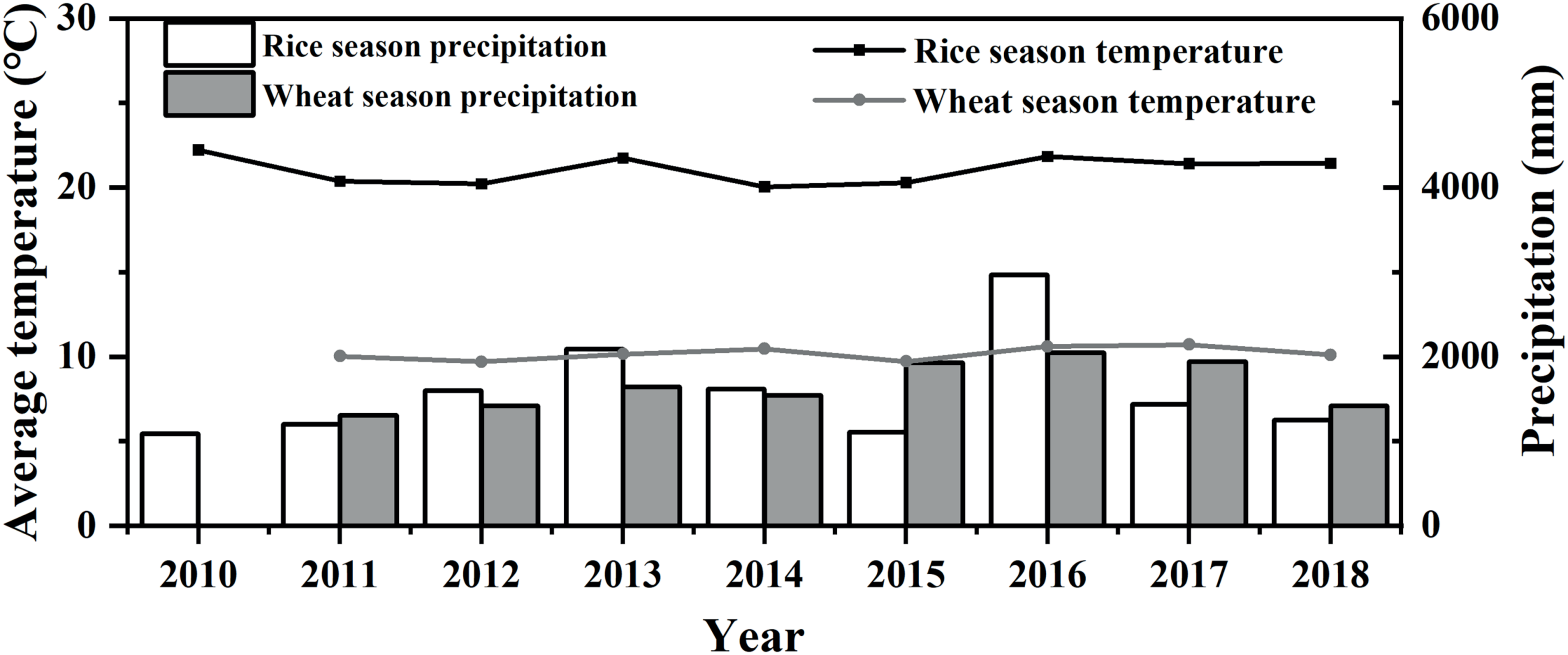
Average temperature and precipitation for the rice and wheat season from 2010–2018. Line: average temperature; Columnar: precipitation.

The experiment was conducted in a rice–wheat rotation system from 2009 to 2018. The three treatments were as follows: *CF* as a control, RBIT only, and RBIT combined with straw incorporation (RBITS). Total N, P and K fertilizer rates for all treatments were the same with 300 kg N ha^−1^, 150 kg P_2_O_5_ ha^−1^, and 240 kg K_2_O in the rice season, respectively, and 225 kg N ha^−1^, 105 kg P_2_O_5_ ha^−1^ and 105 kg K_2_O ha^−1^ in the wheat season, respectively. N rates of the basal fertilizer, tillering fertilizer, spikelets–promoting fertilizer, and spikelets–protecting fertilizer for *CF* and RBIT were 150–75–75–0 kg N ha^−1^ and 120–60–60–60 kg N ha^−1^ in the rice season, respectively, 125–0–100–0 kg N ha^−1^ and 90–45–45–45 kg N ha^−1^ in the wheat season, respectively. P fertilizer rates of the basal fertilizer and jointing fertilizer for *CF* and RBIT were 150–0 kg P_2_O_5_ ha^−1^ and 75–75 kg P_2_O_5_ ha^−1^ in the rice season, respectively, 105–0 kg P_2_O_5_ ha^−1^ and 60–45 kg P_2_O_5_ ha^−1^ in the wheat season, respectively. K fertilizer rates of the basal fertilizer and jointing fertilizer for *CF* and RBIT were 240–0 kg K_2_O ha^−1^ and 120–120 kg K_2_O ha^−1^ in the rice season, respectively, 105–0 kg K_2_O ha^−1^ and 60–45 kg K_2_O ha^−1^ in the wheat season, respectively. Basel fertilizers were applied one day before plowing at the time of rice transplanting and wheat sowing. Tiller fertilizers were applied seven days after rice transplanting and wheat three-leaf stage, respectively. Spikelets–promoting fertilizers were applied in the rice and wheat jointing stage, and then spikelets–protecting fertilizers were applied at the appearance of the inverse 2^nd^ leaf. For RBITS, all rice and wheat straw were returned to the subsequent crop. N, P, and K fertilizer were in the form of urea, triple superphosphate, and potassium chloride, respectively. All treatments were arranged in a randomized block design with three replications, and each plot was 31.5 m^2^. The rice planting density was 13.3 × 30.0 cm, and wheat was sown by broadcasting 225 kg of seed ha^−1^. In each year, the rice variety Wuyunjing 23# (*Oryza sativa* L.) was transplanted by hand in mid–June with a rice planting density of 13.3 × 30.0 cm, and the wheat variety Yangmai 16# (2010–2013) and Yangmai 20 (2014–2018) (*Triticum aestivum* L.) was sown in mid–November at the seed rate of 225 kg ha^−1^. Other agricultural management was carried out following the farmer’s practices. The daily average temperature and precipitation during the rice and wheat seasons from 2010 to 2018 are shown in Figure 1.

### Soil sampling and analyses

#### Collection of soil samples

Due to the small short-term response of total SOC content to fertilizer management, so we measured the total SOC content in the 0–20 cm soil layer annually after the rice harvest beginning in 2014 and continuing for five years through 2018. To clarify the effect of long–term fertilizer management on the SOC pools of different soil layers, we collected soil samples at depths of 0–10 cm and 10–20 cm after the 2018 rice harvest and divided them into two parts. One part was air–dried at room temperature, then ground and sieved to 0.15 mm for analyses of soil bulk SOC and EOC. The other part was sieved to 2 mm and analyzed for MBC, DOC, and HWOC. At the same time, undisturbed soil samples were collected at depths of 0–10 cm and 10–20 cm to measure soil aggregate fractions and their associated C contents.

#### Soil aggregate stability and SOC content in bulk soil and soil aggregate fractions

Soil aggregate fractions were separated using the wet–sieving method (Elliott, 1986). In brief, the following three aggregate size fractions were obtained using a series of sieves: 2–0.25 mm (macro–aggregates), 0.25–0.053 mm (micro–aggregates), and <0.053 mm (silt+clay). Pre-wetted dry soil samples (50 g) were automatically moved 3 cm up and down 30 times per min for 30 min using a soil aggregate analyzer (Model SAA 8052, Shanghai, China). The aggregates that remained on each sieve were then dried at 40°C for 72 h and weighed.

The formula for calculating mass percentage in each particle size aggregate fraction was as follows:


(1) $$m_{a}=\frac{w_{a}}{M}\times 100\%$$

where *m_a_* represents the mass percentage (%) of the a–size aggregate fraction, *w_a_* represents the weight (g) of the a–size aggregate fraction, and *M* is the total weight of all aggregates (g).

The mean weight diameter (MWD) of soil aggregates is a common index used to represent soil aggregate stability and is calculated according to equation 2:


(2) $$MWD=\overset{n}{\underset{a=1}{\sum }}\left(D_{a}\times w_{a}\right)\;$$

where *D_a_* represents the mean diameter (mean of two adjacent sieve diameters) of the a–size aggregate fraction.

Bulk soil and soil aggregates were finely ground (<0.15 mm) to determine SOC content (about 100 mg) with a Flash HT Plus elemental analyzer (Thermo Fisher Scientific, Bremen, Germany).

#### Contents of LOC fractions

MBC was extracted from fresh soil samples (<2 mm, 50 g) by chloroform fumigation and 0.5 M K_2_SO_4_ extraction (Vance et al., 1987) and analyzed using an automated TOC analyzer (Analytik Jena, Germany). The ratio of water and soil was 5:1. The MBC content was calculated using an extraction efficiency coefficient of 0.45 (Jenkinson et al., 2004).

DOC and HWOC were determined from fresh field samples (<2 mm, 10 g) following the procedure of Ghani et al. (2003). The ratio of water and soil was 5:1. The extractions were performed at 25°C for 30 min (DOC) and 80°C for 16 h (HWOC). The DOC and HWOC contents were then analyzed using the automated total organic C (TOC) analyzer (Analytik Jena, Germany).

EOC was determined from dry soil samples (<0.15 mm) following Blair et al. (1995). A soil sample containing about 15 mg C was oxidized with 25 ml 333 mM KMnO_4_ while shaking for 6 h, followed by centrifugation for 10 min at 1000 *g.* The absorbances of the supernatant and standards were read at 565 nm. The change in KMnO_4_ concentration was estimated to calculate the EOC content, assuming that 1 mM KMnO_4_ was consumed in the oxidation of 9 mg of C (Table 1).

**Table 1:** Estimated 9–year cumulative carbon input for each treatment.

Treatment	Root C		Straw C		Total C input
	Wheat	Rice	Wheat	Rice	
*CF*	11.9 b	26.8 c	–	–	38.7 c
RBIT	12.7 a	27.9 b	–	–	40.6 b
RBITS	11.7 b	28.8 a	28.3	37.0	105.7 a

CF: conventional fertilization; RBIT: reduced basal and increased topdressing fertilizer rate; RBITS: RBIT combined with straw incorporation. Different lowercase letters represent significant differences among treatments (p ≤ 0.05). –: no data.

#### C pool management index

The CMPI evaluates the relative potential of agricultural management measures to influence the SOC pool and C sequestration (Zhu et al., 2015). In the present study, the CMPI was used to monitor differences in SOC dynamics between treatments. A higher CMPI value indicated a more sustainable system. The CMPI was calculated using equations 3–6:


(3) CMPI=CPI×LI×100%

where CPI is the C pool index, and LI is the lability index.


(4) $$\mathrm{CPI}=\frac{{\mathrm{SOC}}_{\mathrm{BS}}\;\mathrm{in}\;\mathrm{PF}\;\mathrm{or}\;\mathrm{PFS}\;}{{\mathrm{SOC}}_{\mathrm{BS}}\;\mathrm{in}\;\mathrm{CF}}\times 100\%$$

(5) $$\mathrm{LI}=\frac{E_{\mathrm{EOC}}\;\mathrm{in}\;\mathrm{PF}\;\mathrm{or}\;\mathrm{PFS}}{E_{\mathrm{EOC}}\;\mathrm{in}\;\mathrm{CF}}\;$$

(6) $$E_{\mathrm{EOC}}=\frac{\mathrm{Content of}\;\mathrm{EOC}\;}{\mathrm{SOC}}\;$$

where SOC_BS_ is the SOC content in the bulk soil, and EOC refers to SOC oxidized by KMnO_4_.

#### Yield measurements and estimates of cumulative C input

At the mature stage, grain yields and yield formations were determined by hand–harvesting in each plot; an area of 2.5 m^2^ per plot was harvested in the rice season and 2 m^2^ per plot in the wheat season. The rice and wheat yields were calculated at standard moisture contents of 13.5 and 12.5%, respectively. In the 2016 wheat season, low temperature during the seedling germination stage and poor management caused large–scale weed growth in spring, resulting in an average yield of only 2 t ha^−1^ in 2016. Therefore, to avoid interfering with the research results, the wheat yield and yield formation in 2016 were not counted.

Yield stability was evaluated by comparing the sustainable yield index (SYI) among treatments, which was calculated using equation 7 (Wanjari et al., 2004; Han et al., 2020):


(7) $$\mathrm{SYI}=\frac{Y_{\mathrm{mean}}-Y_{\mathrm{sd}}\;}{Y_{\max}\;}\times 100\%$$

where Y_mean_ is the mean of combined rice and wheat (annual), rice, and wheat yields during 2010–2018, Y_sd_ is the standard deviation of yield, and Y_max_ is the maximum yield over 2010–2018 for each treatment.


(8) $$\mathrm{CV}=\frac{I_{\mathrm{mean}}\;}{I_{\mathrm{sd}}\;}\times 100\%$$

Where CV is the coefficient of variation of year–to–year rice and wheat yield formation parameters. I_mean_ and I_sd_ are the mean and standard deviation of rice and wheat yield formation parameters during 2010–2018.

The 9–year cumulative root–C and straw–C input were estimated (Table 1) using equations 9–11:


(9) $$T_{\mathrm{root or straw}}=\sum W_{\mathrm{root or straw}}+\sum R_{\mathrm{root or straw}}\;$$

(10) $$\sum W_{\mathrm{root or straw}}=\frac{\sum {\mathrm{WB}}_{\mathrm{shoot}}\times {\mathrm{WC}}_{\mathrm{shoot}}}{{\mathrm{WR}}_{\mathrm{shoot}}}\times {\mathrm{WR}}_{\mathrm{root or straw}}$$

(11) $$\sum R_{\mathrm{root or straw}}=\frac{\sum {\mathrm{RB}}_{\mathrm{shoot}}\times {\mathrm{RC}}_{\mathrm{shoot}}}{{\mathrm{RR}}_{\mathrm{shoot}}}\times {\mathrm{RR}}_{\mathrm{root or straw}}$$

where T_root or straw_ is the total cumulative root–C or straw–C input (t C ha^−1^); ∑W_root/straw_ refers to the cumulative root–C or straw–C input (t C ha^−1^) for wheat; ∑R_root or straw_ refers to the cumulative root–C or straw–C input (t C ha^−1^) for rice; ∑WB_shoot_ and WC_shoot_ represent wheat shoot biomass (t ha^−1^) and shoot C content (%), respectively; WR_shoot_ and WR_root or straw_ represent the plant C allocation coefficients of the wheat shoots (grain+straw), roots (including all root–derived material not usually recovered with the roots), and straw, respectively (Table 2); RR_shoot_, RR_root_, and RR _straw_ represent the plant C allocation coefficients for the rice shoots, roots, and straw, respectively. The C content, RR_grain,_ and RR_straw_ were estimated according to the analytical data from 2017 and 2018.

**Table 2:** C content of shoots and relative allocation coefficients of crop C to different parts of wheat and rice.

Crop type	C_shoot_ (%)	Relative crop C allocation coefficients			
		*R_grain_*	*R_straw_*	*R_shoot_*	*R_root_*
Wheat	52	0.32*^#^*	0.48*^#^*	0.80*^#^*	0.20*^#^*
Rice	40	0.36*^##^*	0.36*^##^*	0.72*^###^*	0.28*^###^*

The data marked with # and ### were obtained from the reviews by Bolinder et al. (2007) and Liu et al. (2019). The data marked with ## was calculated based on a rice harvest index of 0.5.

#### SOC sequestration

The SOC stock at a depth of 0–20 cm was calculated using equation 11 (Brar et al., 2015).


(12) SC=SOC×BD×h×10000×0.1

where SC is the SOC stock at a depth of 0–20 cm (Mg ha^−1^), SOC is the SOC content (g kg^−1^) of the plowed layer (0–20 cm), BD is the soil bulk density (kg m^−3^), and h refers to the depth of the plowed layer (0.2 m).

The SOC sequestration (CS) of treatments compared with the initial SOC stock was calculated using equation 13. The sequestrated C rate and C mineralization rate were calculated using equations 14–15.


(13) $$\mathrm{CS}={\mathrm{SC}}_{T}-{\mathrm{SC}}_{\mathrm{initial}}$$

(14) $$\mathrm{CSR}=\frac{\mathrm{CS}}{9}$$

(15) $$\mathrm{CMR}=\frac{\mathrm{CI}}{9}-\mathrm{CSR}$$

where SC_T_ refers to SOC stock after 9 years (Mg ha^−1^), CSR is the C sequestration rate (Mg ha^−1^ year^−1^), CMR is the C mineralization rate (Mg ha^−1^ year^−1^), and CI is an estimate of the total C input over nine years (Mg ha^−1^ year^−1^).

### Data analysis

Statistical analyses of the data were performed using SPSS 26.0 software (IBM Corp., Armonk, NY, USA). All analyses were carried out on the three replicates. A one-way analysis of variance (ANOVA) was performed to analyze the effect of different fertilization management on average yield, SYI, yield compounds, dry matter accumulation, harvest index, different SOC pools, soil aggregate mass distribution, MWD and pH. The least significant difference (LSD) was used to compare mean values between fertilization management. Differences between treatments were significant at *p* ≤ 0.05. Pearson’s correlations were determined among SYI, C input, SOC pool fractions, MWD, and pH, to clarify the relationship between yield stability and soil properties. SOC is usually protected by soil aggregate physically and chemically. Therefore, to determine the main factors affecting SOC, we used redundancy analysis (RDA) to quantify the relationships between the C content of soil aggregate fractions and other parameters (MBC, DOC, HWOC, EOC, C input, MWD, and pH).

## Results

### Yield and yield formation

#### Grain yield

**Figure 2:**
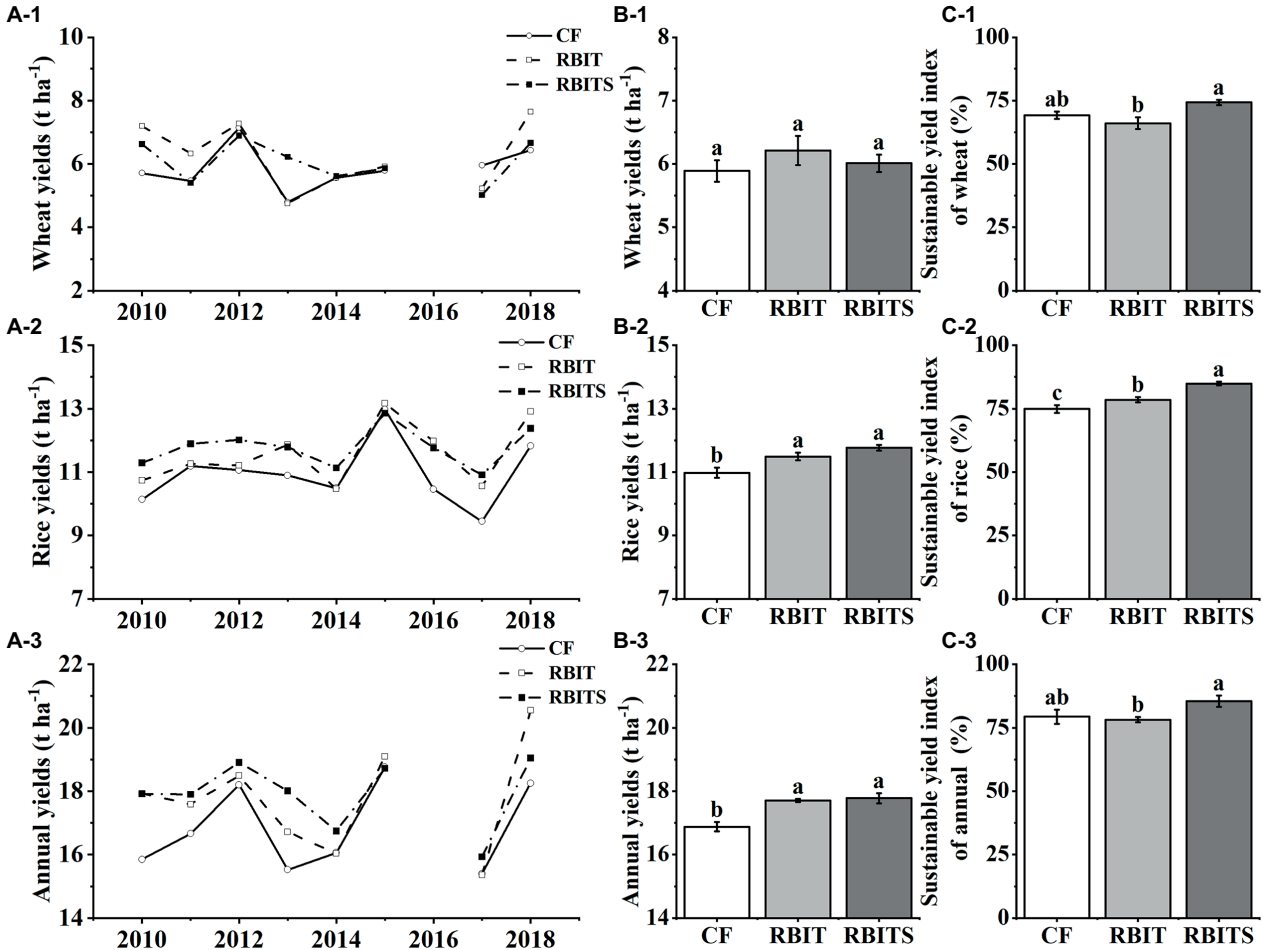
Effects of fertilization management on wheat, rice, and annual yield and their sustainable yield indexes. *CF*: conventional fertilization; RBIT: reduced basal and increased topdressing fertilizer rate; RBITS: RBIT combined with straw incorporation. **(A-C)** Show each year’s yield, the 9–year average yield, and the sustainable yield index, respectively. **(1-3)** Show wheat, rice, and annual data, respectively. Different lowercase letters represent significant differences (*p* ≤ 0.05) among treatments for the same crop type. Error bars are standard error (SE) of the mean.

Over the nine years, wheat yield, rice yield, and annual yield ranged from 4.6–7.6 t ha^−1^, 9.4–13.0 t ha^−1^, and 15.4–20.5 t ha^−1^, respectively (Figure 2A). ANOVA results showed that fertilization management did not affect wheat yield but had a significant effect on the 9–year average rice yield and annual yield (Figure 2B). Compared with the *CF* treatment, the RBIT and RBITS treatments significantly increased the 9–year average rice yield by 4.6 and 6.8% and the annual yield by 4.9 and 5.1%, respectively. However, there was no statistical difference between the RBIT and RBITS treatments.

The wheat, rice, and annual SYIs were 66.0–74.2%, 74.9–84.9%, and 79.4–84.9%, respectively, and they were significantly affected by fertilization management (Figure 2C). Compared with the *CF* treatment, the RBIT treatment did not affect wheat, rice, or annual SYIs, but combined with straw incorporation (RBITS) significantly increased the SYI of rice by 13.4%.

#### Yield components

Fertilization management had no effect on the wheat yield components, and the average No. of panicles and grain weight for rice, but it significantly affected the average No. of spikelets per panicle and seed setting rate for rice (Table 3). Compared with the *CF* treatment, the RBIT and RBITS treatments significantly increased the average No. of spikelets per panicle for rice by 5.5 and 12.3%, respectively, but decreased the seed setting rate by 5.1 and 6.4%, respectively. Among them, the No. of rice spikelets per panicle of the RBITS was higher than that of RBIT.

**Table 3:** Effect of fertilization management on yield components(No. of panicles, No.of spikelets per panicle, grain weight, seed setting rate), dry matter accumulation and harvest index and their coefficients of variation.

Treatments	No. of panicles (m^−2^)		No. of spikelets per panicle		Grain weight (mg)		Seed setting rate (%)		Total dry matter (t ha^−1^)		Harvest index (%)	
	Average	CV	Average	CV	Average	CV	Average	CV	Average	CV	Average	CV
**Wheat**												
*CF*	391.1 ± 34.2 a	24.8	37.0 ± 2.3 a	17.8	41.9 ± 1.6 a	10.5	–	–	10.9 ± 0.5 a	23.5	52.6 ± 1.6 a	14.9
RBIT	405.9 ± 26.1 a	18.2	37.1 ± 2.3 a	17.3	42.4 ± 1.9 a	12.8	–	–	11.7 ± 0.4 a	15.5	53.6 ± 1.8 a	15.2
RBITS	386.8 ± 21.7 a	15.9	38.4 ± 2.1 a	15.4	42.0 ± 1.6 a	10.5	–	–	11.0 ± 0.3 a	12.5	51.3 ± 1.8 a	15.8
**Rice**												
*CF*	327.1 ± 7.9 A	12.2	123.4 ± 2.3 C	9.5	30.4 ± 0.2 A	3.9	89.9 ± 1.0 A	5.7	19.1 ± 0.3 B	8.1	49.4 ± 0.9 A	8.5
RBIT	344.5 ± 6.6 A	10.0	130.3 ± 1.5 B	6.1	30.3 ± 0.3 A	5.5	85.4 ± 1.3 B	8.0	19.9 ± 0.3 A	6.5	50.1 ± 0.8 A	8.3
RBITS	337.5 ± 7.4 A	11.5	138.7 ± 1.1 A	4.1	30.3 ± 0.3 A	5.2	84.2 ± 1.5 B	9.1	20.6 ± 0.2 A	5.8	49.4 ± 0.7 A	6.7

CF: conventional fertilization; RBIT: reduced basal and increased topdressing fertilizer rate; RBITS: RBIT combined with straw incorporation. MWD: mean weight diameter. Different lowercase and uppercase letters represent significant differences in yield components of wheat and rice, respectively (P ≤ 0.05). Data: mean ± standard error (SE); −: no data.

In terms of CVs, wheat yield components were higher than rice, and they were ranked No. of panicles (15.9–24.8%) > No. of spikelets per panicle (15.4–17.8%) > grain weight (10.5–12.8%) for wheat and No. of panicles (10.0–12.2%) > seed setting rate (5.7–9.1%), No. of spikelets per panicle (4.1–9.5%) > grain weight (3.9–5.5%) for rice (Table 3). Compared with the *CF* treatment, the RBIT and RBITS treatments reduced the CVs of No. of panicles and No. of spikelets per panicle but increased the CVs of grain weight and seed setting rate; similar trends were observed in rice and wheat.

#### Dry matter accumulation and harvest index

Fertilization management significantly affected the average dry matter accumulation for rice, showing RBITS (20.6 t ha^−1^) > RBIT (19.9 t ha^−1^) > *CF* (19.1 t ha^−1^), but it had no effect on the average dry matter accumulation for wheat and harvest index for rice and wheat (Table 3).

In terms of CVs, dry matter accumulation (12.5–23.5%) and harvest index (16.3–18.8%) for wheat were higher than for rice (5.8–8.1%, 6.7–8.5%). The CVs of dry matter accumulation and harvest index among the treatments, except for the harvest index for wheat, showed a consistent trend, RBITS > RBIT > *CF* (Table 3).

### SOC sequestration

**Figure 3:**
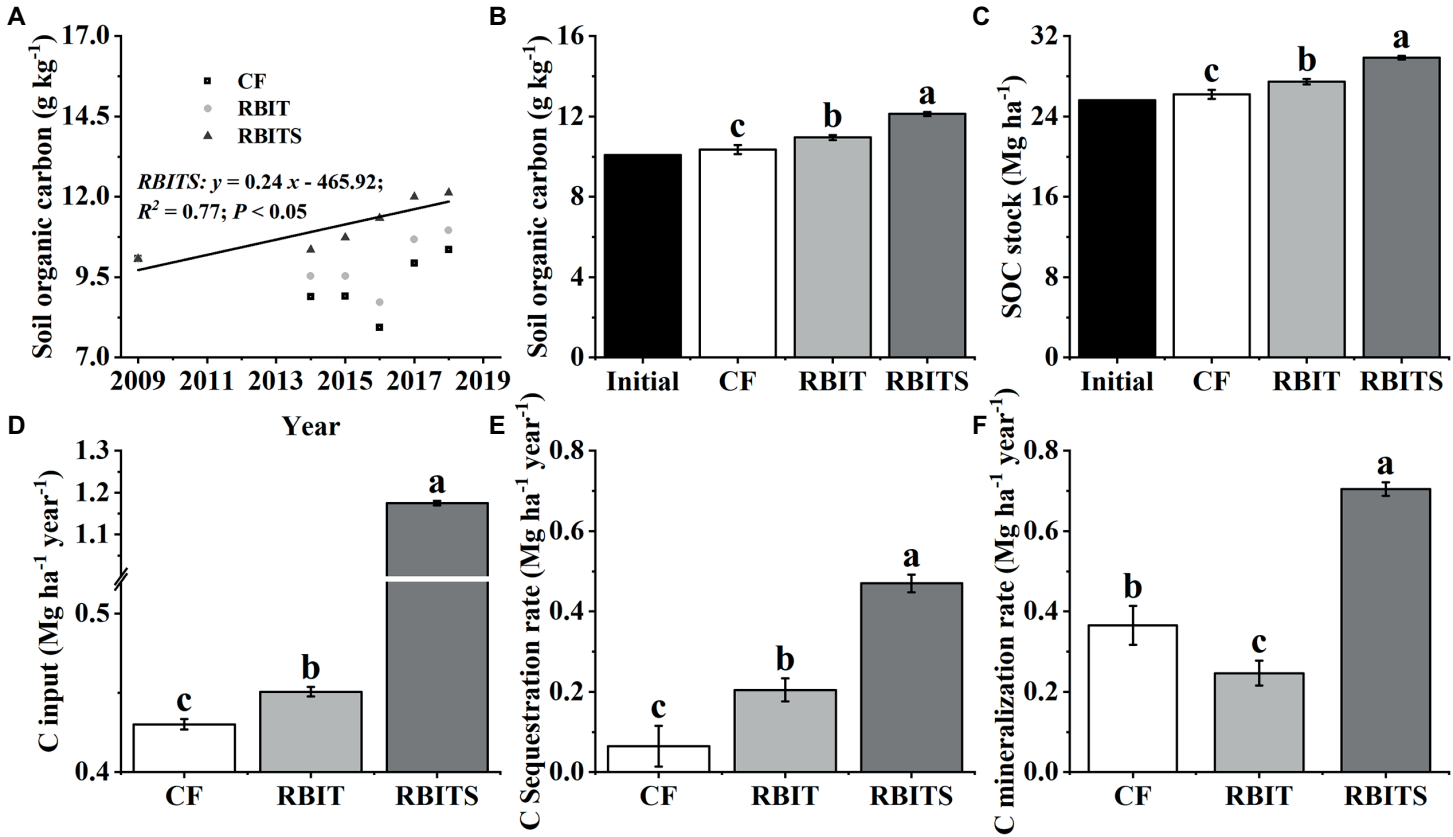
Effect of fertilization management on SOC dynamics **(A)**, SOC in 2018 **(B)**, SOC stock **(C)**, C input **(D)**, C sequestration rate **(E)**, and C mineralization rate **(F)** in the bulk soil (0–20 cm). *CF*: conventional fertilization; RBIT: reduced basal and increased topdressing fertilizer rate; RBITS: RBIT combined with straw incorporation. Different lowercase letters represent significant differences (*p* ≤ 0.05) among treatments for the same crop type. Error bars are standard error (SE) of the mean.

Among the treatments, only bulk soil SOC (0–20 cm) (*R^2^* = 0.77, *p* < 0.05) in the RBITS treatment showed a significant trend of increasing over time (Figure 3A). After nine years, the content and stock of SOC in the 0–20 cm layer of the *CF* (10.3 g kg^−1^, 26.2 Mg ha^−1^), RBIT (11.0 g kg^−1^, 27.5 Mg ha^−1^), and RBITS (12.1 g kg^−1^, 29.8 Mg ha^−1^) treatments were higher than their initial values (10.1 g kg^−1^, 25.6 Mg ha^−1^) (Figures 3B,C). Statistical differences were observed between the *CF* treatment and the RBIT treatment with and without straw incorporation.

The estimated values of C input, C sequestration rate, and C mineralization rate in the 0–20 cm layer were 0.43–1.18 Mg ha^−1^ year^−1^, 0.06–0.47 Mg ha^−1^ year^−1^, and 0.25–0.71 Mg ha^−1^ year^−1^, respectively (Figures 3D–F). Compared with the *CF* treatment, the RBIT and RBITS treatments significantly increased C input and C sequestration rate by 4.8, 173.1, 216.5, and 627.6%, respectively. In terms of the C mineralization rate, the RBIT treatment significantly decreased by 32.6%, but when combined with straw incorporation (RBITS), it increased by 92.8%.

### SOC pools

#### Aggregate–associated C

**Figure 4:**
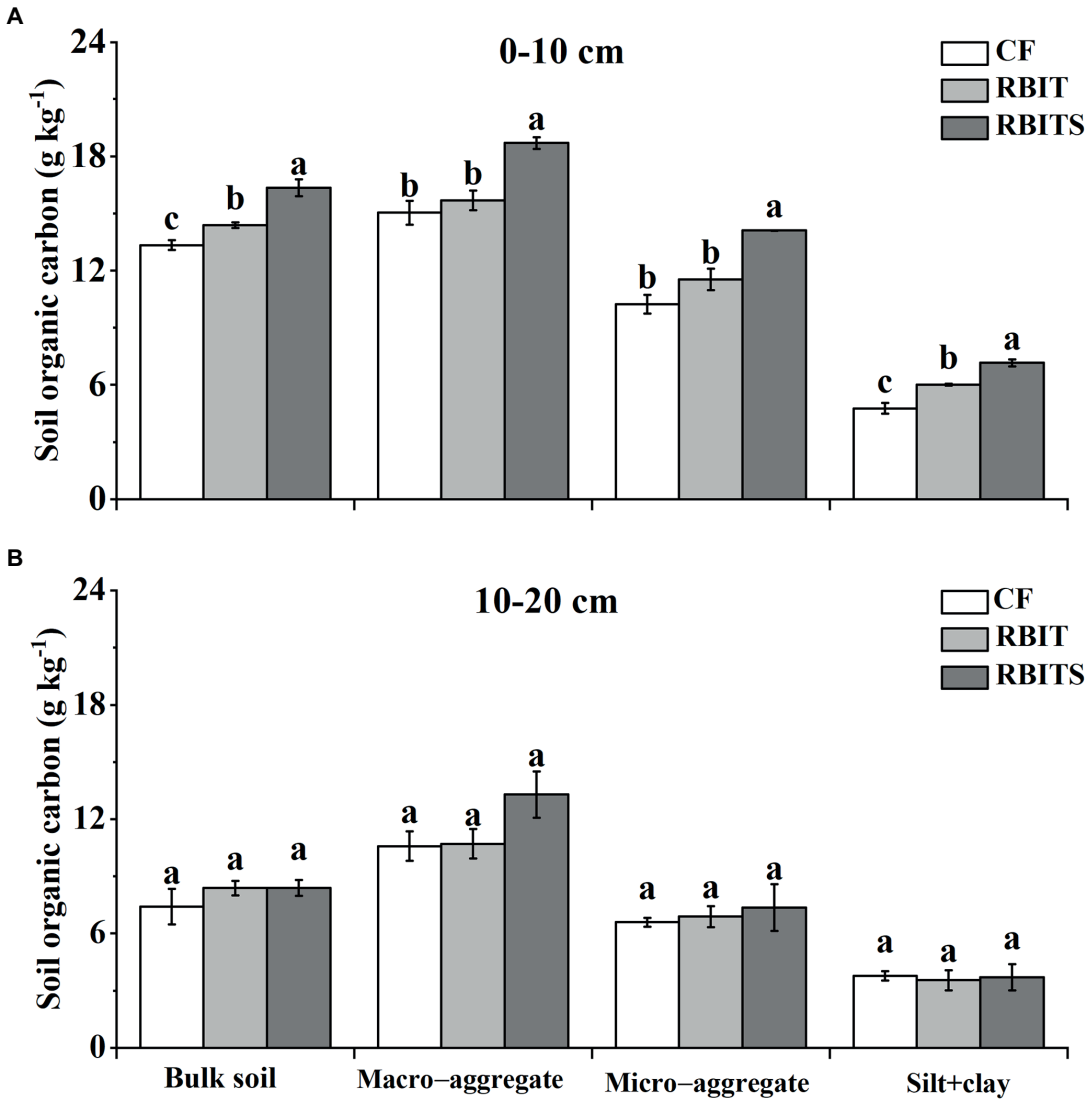
Effect of fertilization management on the SOC content in the bulk soil and aggregate fractions at depths of 0–10 cm **(A)** and 10–20 cm **(B)** after nine years. *CF*: conventional fertilization; RBIT: reduced basal and increased topdressing fertilizer rate; RBITS: RBIT combined with straw incorporation. Different lowercase letters represent significant differences (*p* ≤ 0.05) among treatments in each graph and for each fraction. Error bars are standard error (SE) of the mean.

Figure 4 shows that fertilization management significantly affected the SOC content of bulk soil and aggregate fractions in the 0–10 cm layer but had no effect in the 10–20 cm layer. In the 0–10 cm layer, compared with the *CF* treatment, the RBIT treatment significantly increased the SOC content of bulk soil and silt+clay by 8.35 and 25.0% but did not markedly affect the macro– and micro–aggregate fractions; when RBIT combined with straw incorporation (RBITS), it significantly increased SOC levels in the bulk soil and macro–aggregate, micro–aggregate, and silt+clay fractions by 22.5, 24.4, 37.8, and 50.1%, respectively.

#### LOC fractions

**Figure 5:**
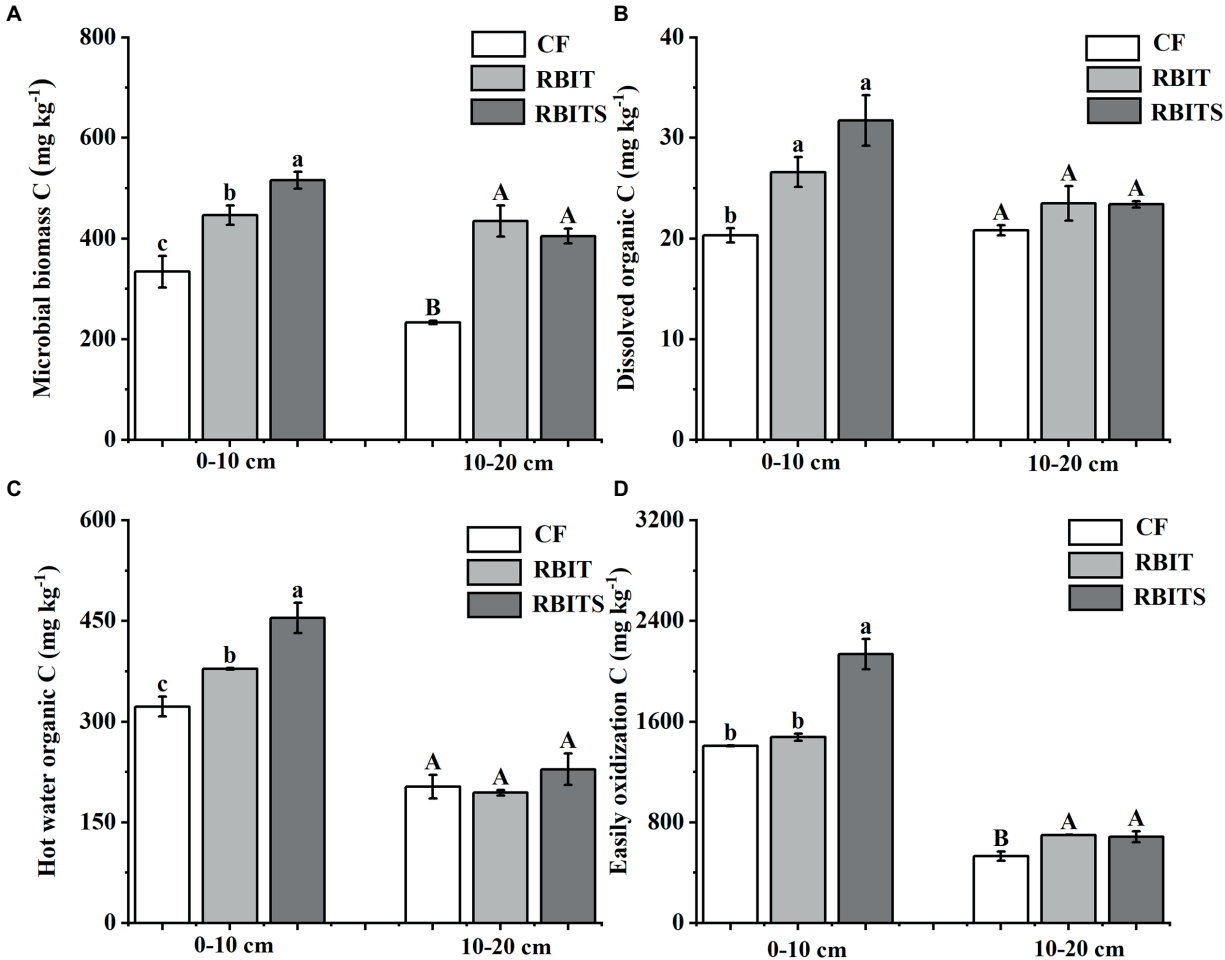
Effect of fertilization management on the content of microbial biomass C **(A)**, dissolved organic C **(B)**, hot water organic C **(C)**, and easily oxidizable C **(D)** at depths of 0–10 cm and 10–20 cm after nine years. *CF*: conventional fertilization; RBIT: reduced basal and increased topdressing fertilizer rate; RBITS: RBIT combined with straw incorporation. MBC: microbial biomass carbon; DOC: dissolved organic C; HWOC: hot water organic C; EOC: easily oxidizable C. The different lowercase and uppercase letters represent significant differences (*p* ≤ 0.05) among treatments at depths of 0–10 cm and 10–20 cm, respectively. Error bars are standard error (SE) of the mean.

**Figure 6:**
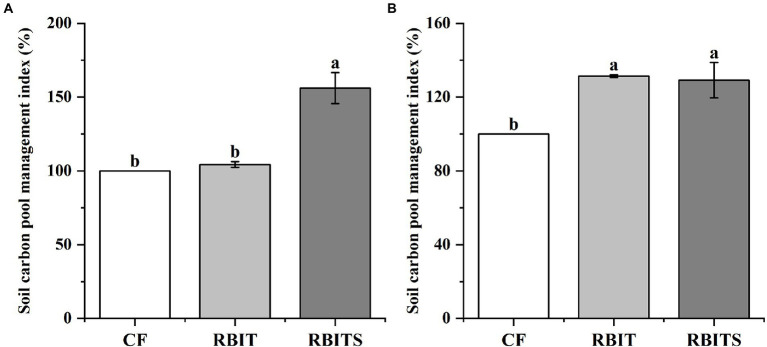
Effect of fertilization management on CMPI at depths of 0–10 cm **(A)** and 10–20 cm **(B)** after nine years. *CF*: conventional fertilization; RBIT: reduced basal and increased topdressing fertilizer rate; RBITS: RBIT combined with straw incorporation. The different lowercase letters represent significant differences (p ≤ 0.05) among treatments in each soil depth. Error bars are standard error (SE) of the mean.

LOC pool fractions in the two soil depths responded differently to fertilization management (Figure 5). In the 0–10 cm layer, the contents of MBC (446.5, 515.7 mg kg^−1^), HWOC (378.5, 454.8 mg kg^−1^), and DOC (26.6, 31.72 mg kg^−1^) in the RBIT alone or in combination with straw incorporation (RBITS) were higher than in the *CF* treatment (334.1 mg kg^−1^, 322.3 mg kg^−1^, and 20.3 mg kg^−1^). The RBITS treatment had the highest content of EOC among the treatments. In the 10–20 cm layer, fertilization management significantly affected the contents of MBC and EOC only. The RBIT and RBITS treatments increased MBC by 86.7 and 73.8%, and EOC by 31.6 and 29.4%, respectively, compared with the *CF* treatment. Furthermore, application of RBIT alone or in combination with straw incorporation (RBITS) produced a higher CMPI at both soil depths compared with the *CF* treatment, with increases of 4.3–31.1% and 29.2–31.4% at depths of 0–10 cm and 10–20 cm, respectively (Figure 6).

### Soil aggregate stability and pH

The mass proportions of soil aggregate fractions were ranked macro–aggregates > micro–aggregates > silt+clay (Table 4). Compared with the *CF* treatment, the RBIT treatment produced no change in soil aggregate mass proportions at the two soil depths, but when combined with straw incorporation (RBITS), it had a significant effect on the mass proportions of macro–aggregate and micro–aggregate fractions in the 0–10 cm layer, increasing the macro–aggregate fraction by 13.9% and decreasing the micro–aggregate fraction by 36.7%. In terms of MWD, only the 0–10 cm layer showed significant differences among treatments, which were ranked RBITS (89.5 mm) > *CF* (80.3 mm) and RBIT (77.0 mm). However, there were no differences in pH among treatments at either soil depth (Table 4).

**Table 4:** Effect of fertilization management on soil aggregate mass distribution, mean weight diameter and pH at depths of 0–10 cm **(A)** and 10–20 cm **(B)** after nine years.

Treatments	Aggregate mass proportion (%)			MWD (mm)	pH (soil/water: 1/2.5)
	2–0.25 mm	0.25–0.053 mm	<0.053 mm		
0–10 cm					
*CF*	67.8 ± 1.6 b	24.7 ± 0.9 a	7.4 ± 0.7 a	80.3 ± 1.6 b	5.5 ± 0.2 a
RBIT	64.6 ± 2.6 b	27.2 ± 2.5 a	8.2 ± 0.4 a	77.0 ± 2.6 b	5.3 ± 0.0 a
RBITS	77.3 ± 1.7 a	15.6 ± 1.7 b	7.1 ± 0.5 a	89.5 ± 1.7 a	5.5 ± 0.0 a
10–20 cm					
*CF*	55.0 ± 9.2 A	34.9 ± 6.0 A	10.1 ± 3.5 A	67.5 ± 5.1 A	6.4 ± 0.1 A
RBIT	54.2 ± 7.2 A	35.2 ± 4.7 A	10.6 ± 2.9 A	66.6 ± 6.0 A	6.1 ± 0.1 A
RBITS	51.8 ± 4.9 A	39.4 ± 3.9 A	8.8 ± 1.6 A	64.4 ± 5.2 A	6.2 ± 0.1 A

CF: conventional fertilization; RBIT: reduced basal and increased topdressing fertilizer rate; RBITS: RBIT combined with straw incorporation. MWD: mean weight diameter. Different lowercase and uppercase letters represent significant differences (P ≤ 0.05) among treatments in the 0–10 cm and 10–20 cm layers, respectively. Data: mean ± standard error (SE).

### Relationships between yield stability and soil properties

**Figure 7:**
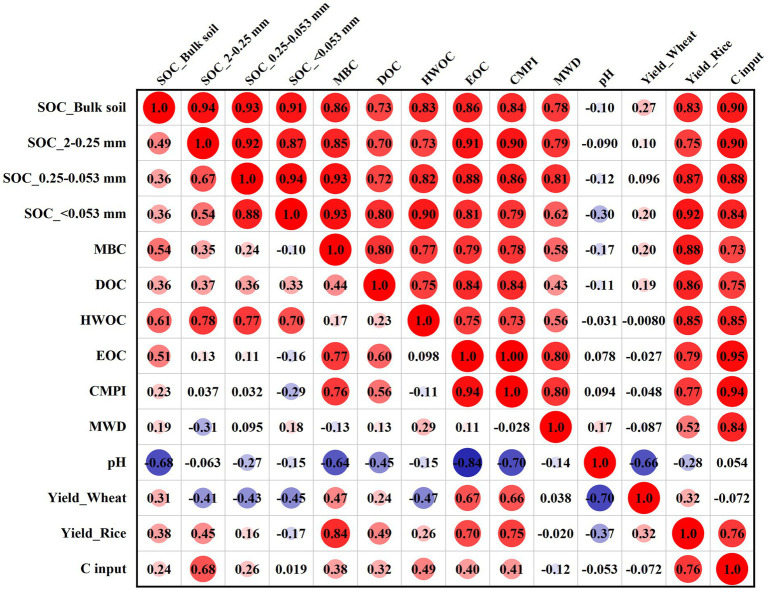
Correlation analysis between the SYI of wheat and rice and soil parameters at depths of 0–10 cm (upper triangular matrix) and 10–20 cm (lower triangular matrix). MBC: microbial biomass carbon; DOC: dissolved organic C; HWOC: hot water organic C; EOC: easily oxidized C; CMPI: C pool management index; MWD: mean weight diameter. SYI: sustainable yield index.

Correlation analysis showed that the SYI of wheat and rice had a positive correlation with SOC fraction content, CMPI, and MWD in the 0–10 cm layer, and these relationships were stronger for rice (Figure 7). Significant positive correlations between different SOC pools were observed only in the 0–10 cm layer. Furthermore, MWD was significantly correlated with SOC in the bulk soil and macro–aggregate and micro–aggregate fractions, and with EOC and C input in the 0–10 cm layer, but no significant relationships were observed in the 10–20 cm layer.

**Figure 8:**
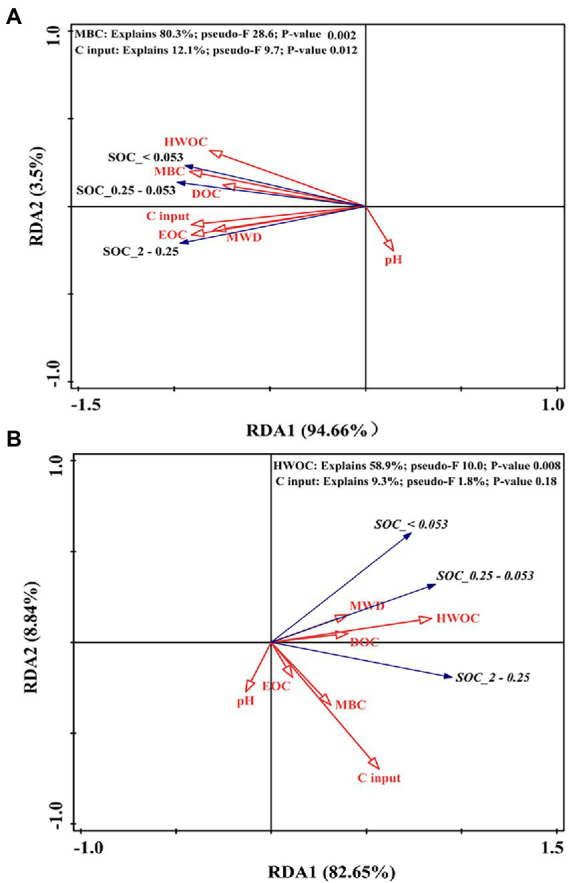
RDA plot showing the associations between the SOC content in soil aggregate fractions and different pools of organic C (MBC, DOC, HWOC, and EOC), C input, pH, and MWD at depths of 0–10 cm **(A)** and 10–20 cm **(B)**. MBC: microbial biomass carbon; DOC: dissolved organic C; HWOC: hot water organic C; EOC: easily oxidized C; MWD: mean weight diameter.

For the soil aggregate–associated C in the 0–10 cm layer, the first axis accounted for 94.7% of the overall variance, and the second axis accounted for 3.5% (Figure 8A); MBC (*F* = 28.6, *p* = 0.002) and C input (*F* = 9.7, *p* = 0.012) were the main parameters that affected soil aggregate–associated C, accounting for 80.3 and 12.1% of the total variance, respectively. In the 10–20 cm layer, the first and second axes accounted for 74.4 and 8.8% of the variance (Figure 8B). HWOC (*F* = 10.0, *p* = 0.008) was the main parameter that affected the soil aggregate–associated C content, explaining 58.9% of the total variance.

## Discussion

### RBIT with straw incorporation and increased crop yields in the rice–wheat system

With population growth and urbanization, increasing the grain yield per unit area is important for meeting future high food demands (Zhang et al., 2015; Hemathilake and Gunathilake, 2022). In our study, 9–year average rice yields of RBIT with/without straw incorporation in a rice–wheat system are consistent with previous studies (Khurana et al., 2008; Fan et al., 2009; Kamiji et al., 2011; Ercoli et al., 2013; Sui et al., 2013; Moe et al., 2014; Chen et al., 2015; Xu et al., 2015; Wang et al., 2019; Wood et al., 2020; Ye et al., 2022), in which found that higher grain yield and N use efficiency were obtained with reduced N application before seeding. On the one hand, spikelets formation begins at the beginning of tillering and the number of spikelets is determined at the beginning of stem elongation (Mohapatra and Sahu, 2022). The yield advantage was related to a higher number of kernels per spike, resulting from a higher number of fertile spikelets per spike (Kamiji et al., 2011; Ercoli et al., 2013; Sui et al., 2013; Chen et al., 2015; Wang et al., 2019; Ye et al., 2022). Reducing N fertilizer rate in the early growth stage could limit the production of ineffective tillers, forming a healthy population (Myint et al., 2009; Zhang et al., 2011; Moe et al., 2014; Ye et al., 2022). Providing sufficient nutrients at the stem elongation stage could promote the differentiation of spikelets and reduce their degradation, thereby obtaining high No. of spikelets per panicle (Kamiji et al., 2011; Zhang et al., 2011; Sui et al., 2013). Similar results were observed in our 9-year field experiment. On the other hand, N is important for leaf chlorophyll, and fertilizer N application could improve leaf chlorophyll content and the photosynthetic capacity of crops (Zhang et al., 2021). Field experiments reported that the high yield of the crop was mainly attributable to the higher total and post-anthesis dry matter accumulation (Zhang et al., 2011, 2021; Ye et al., 2022). This study observed that RBIT had a high SPAD valve of 1^st^–4^th^ leaf from the top (Supplementary Table S1) and photosynthetic rate of sword leaf (Supplementary Table S2) at the rice heading stage, thereby promoting the synthesis of photosynthetic products.

Abundant mineral elements in returned straw provide nutrients for crop growth and spikelet differentiation (Huang et al., 2013; Li et al., 2018). Previous studies observed that straw incorporation could immobilize fertilizer–derived N from previous and current seasons by improving soil quality and through the straw itself (Cao et al., 2018; Zheng et al., 2019). This immobilized N is released at the high nutrient demand stage (after the jointing stage) (Cao et al., 2018; Zheng et al., 2019), making obtain more No. of spikelets per panicle (Huang et al., 2013). In our study, straw incorporation had a high No. of spikelets per panicle but had a slightly negative effect on No. of panicles. This result was because the initial decomposition of straw could release phytotoxic substances (e.g., organic acids) and decrease soil available N content with high microbial N immobilization, thereby inhibiting rice tillering (Sharma and Prasad, 2008; Olk et al., 2009; Wei et al., 2019). Thus, the positive effects of straw incorporation on rice were possibly offset by its negative effects, leading to an ineffective yield increase.

However, negative effects of RBIT with/without straw incorporation on the seed setting rate for rice was possibly due to intensified competition for resources (e.g., space, nutrients, carbohydrates) between spikelets. Previous studies found a negative feedback effect between the number of spikelets and grain filling (Kamiji et al., 2011; Sui et al., 2013). Therefore, high yield strategies should pay greater attention to the intense source and smooth translocation for enlarging effective sink–filling ability in rice–wheat systems.

It is worth noting that RBIT with/without straw incorporation had no effect on wheat yield, which was in agreement with previous studies (Garrido-Lestache et al., 2005; Pampana and Mariotti, 2021). Mariana et al. (2003) found that N applications at tillering permit the highest wheat yields. Some studies demonstrated that the early season N environment had a large influence on N partitioning at maturity, whereas N applied at anthesis had little effect on N partitioning and was allocated more efficiently to wheat grain (Melaj et al., 2003; Wang et al., 2018). However, Shi et al. (2012) found that topdressing could obtain a high wheat yield due to giving better root growth and increasing plant N uptake. Zhang et al. (2021) found that increasing topdressing N fertilizer under water-saving irrigation conditions could promote antioxidant enzyme activity and the remobilization of photosynthate after anthesis to increase wheat grain yield. In conclusion, this discrepancy may be attributed partly to variations in climatic conditions (i.g., rainfall and temperature), and soil residual N content prior to sowing (Melaj et al., 2003; Garrido-Lestache et al., 2005; Myint et al., 2009; Pampana and Mariotti, 2021).

### RBIT with straw incorporation and crop yield stability in the rice–wheat system

SYI has been used to evaluate the sustainability and stability of crop production under climate change, and a lower value of SYI indicates a more unstable system (Wanjari et al., 2004; Han et al., 2020; Shi et al., 2022). In our study, the SYI of rice (74.9–84.9%) was higher than that of wheat (66.0–74.2%), in agreement with Han et al. (2020), who reported that the SYI of wheat and rice were 50–69% and 39–53%, respectively. Those results suggested that wheat growth was more vulnerable to year–to–year weather change (Fatima et al., 2022). Under the effects of climate change, the microclimate of the rice root growth environment can be protected by the water layer, whereas that of wheat is not. For upland rainfed wheat, mineralization of fertilizers and soil N depends on rainfall (namely soil water content) and temperature, affecting the establishment of tillers, the differentiation and formation of spikelets, and the synthesis of photosynthate (Garrido-Lestache et al., 2005; Homyak et al., 2017; Pampana and Mariotti, 2021). Indeed, in our study, the CVs of wheat panicle number (15.9–24.8%), spikelets per panicle (15.4–17.8%), dry matter accumulation (12.5–23.5%) were higher than that of grain weight (10.5–12.8%) and harvest index (14.9–15.8%).

Our experiment found that the application of RBIT did not affect crop yield stability in the rice–wheat system, but RBIT combined with straw incorporation helped stabilize rice and wheat yield, especially rice, which was in agreement with previous studies (Qaswar et al., 2019; Shi et al., 2022). These results might be attributed to alleviating the negative effect of climate change on No. of panicles, No of spikelets per panicle and dry matter accumulation. Tillering, panicle differentiation and material synthesis depend on the nutrient status of crops (Melaj et al., 2003; Chen et al., 2015; Wang et al., 2018; Ye et al., 2022). Our results showed that the year–to–year fluctuation of the SPAD value of the 1^st^–4^th^ leaf from the top at heading in the RBITS treatment was lower than that of the RBIT and *CF* treatments (Supplementary Figure S1). This finding implied that RBITS could stabilize the N status of crops, which might improve crop resistance to climate change. Strong root system and nutrient (i.g., N, K, Si) supply under straw incorporation (Li et al., 2018; Yan et al., 2018; Wei et al., 2019) might provide a favorable condition for stabilizing the nutrient status of crops and resisting abiotic stress (i.g., climate change). Indeed, Pan et al. (2016) showed that N fertilizer application could compensate for the negative effects of shading on photosynthesis and root morphologies of rice. A review summarized Si–mediated abiotic and biotic stress tolerance mechanisms by scavenging the reactive oxygen species and regulating different metabolic pathways (Ranjan et al., 2021). K application also had similar effects to Si application in alleviating abiotic stresses, contributing to the accumulation of photosynthetic products and N uptake (Sustr et al., 2019; Wang et al., 2020). These results suggested that RBITS might improve the physiological and biochemical mechanism of crop stress resistance for alleviating the negative effect of climate change and stabilizing crop yield. However, the effects of RBITS practice on the physiological/biochemical mechanisms underlying crop stability warrant further research.

### Effect of RBIT and straw incorporation on SOC sequestration

The magnitude and direction of SOC sequestration are highly dependent on the quantity and quality of exogenous organic matter (Xu et al., 2020; Angst et al., 2021; Mohamed et al., 2021). In our experiment, root residues and root exudates were the main sources of soil organic matter input in the *CF* and RBIT treatments. RBIT application had a high SOC stock owing to vigorous crop growth and low SOC mineralization rate. A meta-analysis reported that increasing the splitting frequency of fertilizer N application and decreasing basal N fertilizer could reduce soil reactive N losses (Xia et al., 2018). ^15^N tracer showed that RBIT application could increase the residual amount of panicle fertilizer N in the soil and decrease fertilizer N loss (Xu et al., 2015; Wang et al., 2018; Zheng et al., 2019; Ye et al., 2022). In this study, total N, P, and K contents after nine years of RBIT application were higher than the initial value and the value in the *CF* treatment (data not shown), indicating that this practice promotes the soil retention of fertilizer–derived nutrients and reduces the mineralization of soil organic matter.

It is widely acknowledged that there are two pathways by which microbes promote soil C turnover: one is catabolism, releasing CO_2_ to the atmosphere from the mineralization of organic matter; the other is anabolism, sequestering C (such as the formation of stabilized C, humic acid) by the microbial “C pump” that processes fresh C inputs. In previous work, the relative strength of the two processes was closely related to C utilization efficiency (CUE) (Chen et al., 2020). When straw was removed, DOC input from rhizodeposition was the primary C source for microbial growth (Zhu et al., 2017). Root–derived C was regarded as a more efficient C source for the formation of stable SOC than aboveground residues (Kätterer et al., 2011). Indeed, in our study, DOC, MBC, and humic acid in the surface layer (data not shown) in the RBIT treatment were higher than that of the CT treatment. This result suggested that the microbial anabolism pathway was stronger than the catabolism pathway in the RBIT treatment, thus promoting humification. Christopher and Lal (2007) reported similar results in which optimized N fertilizer management could foster humus formation. Some studies have indicated that microbial necromass was associated with clay minerals, forming bio–recalcitrant organic compounds (Kätterer et al., 2011; Angst et al., 2021; Sokol et al., 2022). Our results showed that nine years of RBIT application positively affected silt+clay associated–C in the surface layer, and surface silt+clay associated–C was the most closely related to MBC, suggesting that RBIT helped to increase the stable organic C pool. Therefore, the long–term application of RBIT contributes to stabilizing SOC pools, decreasing C emissions under climate change, and slowing the rate of global warming.

Straw incorporation as a globally protective tillage practice can improve the quantity and quality of SOC (Liu et al., 2014; Xia et al., 2018; Xu et al., 2020; Mohamed et al., 2021; Shi et al., 2022), as seen in the results of this study. This effect was due to residual plant input (Angst et al., 2021). In contrast to the C sequestration of RBIT, aboveground residues rich in easily decomposed organic matter (such as glucose and cellulose) were added to the soil, triggering the metabolism of microorganisms in the catabolism pathway and producing more CO_2_ (Liu et al., 2014; Chen et al., 2020; Angst et al., 2021). In our study, the mineralization rate in the RBITS treatment was 0.9–fold and 1.3–fold that of *CF* and RBIT, implying that straw incorporation enhanced soil C emission (Liu et al., 2014; Xia et al., 2018). If this is true, how do we reduce soil C emissions when the straw is returned to the field? In this study, the negative effect of RBIT on the mineralization rate of SOC suggests that adjusting fertilization patterns under conditions of straw incorporation can reduce soil C emissions and synergize the effective use of nutrients, thereby reducing the risks of climate warming and environmental pollution.

### Effect of RBIT and straw incorporation on soil physical properties

In our study, continuous application of RBIT caused no change in soil aggregate structure, but combined with straw incorporation could increase macro–aggregate mass percentage and MWD in the surface layer. Previous studies have shown similar results in which the positive effect of straw incorporation on soil structure was greater than that of inorganic fertilizer (Liu et al., 2014; Jin et al., 2020). This result may be attributable to the cementing effect of plant–derived compounds (such as carbohydrates), roots, and hyphae to fine particles. Some studies have reported that straw incorporation promoted strong root systems and high microbial biomass (Liu et al., 2014; Xia et al., 2018; Yan et al., 2018; Chen et al., 2020). In our study, positive correlations between MWD and SOC pool fractions and C input in the surface layer implied that stabilized soil structure in the RBITS treatment might limit organic matter decomposition and increase SOC storage.

Furthermore, no significant reduction in soil pH after nine years of RBIT application implies that long–term application may prevent soil acidification, which may be occurred through the substantial removal of base cations under conventional high–yield management (Zeng et al., 2017). Previous studies reported that straw incorporation could help to reduce soil acidification by returning base cations in crop straw to the soil (Zeng et al., 2017; Li et al., 2018). Similar results were also observed in this study. Therefore, long–term straw incorporation as a soil amendment can reduce soil acidification caused by the excessive application of inorganic N fertilizer.

### Yield stability and soil properties

In our study, the SYIs of rice had positive relationships with SOC fractions and MWD in the surface layer, which was consistent with previous research (Wanjari et al., 2004; Han et al., 2020). This finding implied that improved soil quality could benefit the stability and sustainability of rice production (Wanjari et al., 2004; Qaswar et al., 2019; Han et al., 2020; Rubio et al., 2021; Shi et al., 2022). However, a weaker positive relationship between wheat yield stability and soil parameters suggested that improved wheat adaptability to climate change might require the selection and breeding of varieties with high tolerance to low temperatures and waterlogging injury based on appropriate cultivation practices.

## Conclusion

A 9–year field experiment showed that RBIT positively affected rice yield and annual yield in rice–wheat systems but did not significantly affect yield stability. RBIT combined with straw incorporation stabilized rice yield owing to low year–to–year fluctuations in the No. of panicles, No. of spikelets per panicle, and dry matter accumulation. Long–term application of RBIT contributed to SOC sequestration due to high root C input and low C mineralization rate. When combined with straw incorporation, RBIT further improved SOC stock. Long–term RBIT application significantly increased silt+clay–associated C, MBC, DOC, and HWOC in the surface soil but not in the subsurface soil, and RBIT with straw incorporation resulted in the greatest SOC pool fractions and the most stable soil structure. Correlation analysis indicated that wheat and rice yields were more stable with the improvement in SOC, its fractions, and soil structure in the surface layer. Our findings suggest that the long–term application of RBIT with straw incorporation can improve sustainable rice production and SOC sequestration in rice–wheat systems.

## Data availability statement

The original contributions presented in the study are included in the article/Supplementary material, further inquiries can be directed to the corresponding author.

## Author contributions

JZ and JW contributed to the conception and design of the study. JZ, YZ, and LX organized the database. JZ performed the statistical analysis and wrote the first draft of the manuscript. All authors contributed to the article and approved the submitted version.

## Funding

The research was supported by the National Key Research and Development Program of China (2017YFD0301203, 2017YFD0300100, and 2018YFD0300803) and the Jiangsu Agriculture Science and Technology Innovation Fund [JASTIF; CX(21)1009].

## Conflict of interest

The authors declare that the research was conducted in the absence of any commercial or financial relationships that could be construed as a potential conflict of interest.

## Publisher’s note

All claims expressed in this article are solely those of the authors and do not necessarily represent those of their affiliated organizations, or those of the publisher, the editors and the reviewers. Any product that may be evaluated in this article, or claim that may be made by its manufacturer, is not guaranteed or endorsed by the publisher.
