# Supplementary material for: Reduced basal and increased topdressing fertilizer rate combined with straw incorporation improves rice yield stability and soil organic carbon sequestration in a rice–wheat system
Source: Front Plant Sci. 2022 Aug 26;13:964957. doi: 10.3389/fpls.2022.964957 (PMC9459092; doi:10.3389/fpls.2022.964957)
Supplement: Supplementary file 1 [file Data_Sheet_1.docx]

**Supplementary material**

**Table S1 Effect of fertilization management on the SPAD value of 1^st^–4^th^ leaf from the top at rice heading stage and their coefficients of variation**

| Treatment | 1^st^ leaf | | 2^nd^ leaf | | 3^rd^ leaf | | 4^th^ leaf | |
| --- | --- | --- | --- | --- | --- | --- | --- | --- |
|  | Average | CV | Average | CV | Average | CV | Average | CV |
| CF | 46.6±0.6 b | 5.7 | 45.6±0.6 b | 6.1 | 45.7±0.5 b | 6.0 | 45.8±0.6 b | 6.9 |
| BRTI | 49.4±0.4 a | 4.1 | 47.8±0.3 a | 2.8 | 47.4±0.4 a | 3.9 | 47.8±0.4 b | 4.6 |
| BRTS | 48.8±0.3 a | 3.1 | 48.8±0.2 a | 2.1 | 47.0±0.3 a | 2.8 | 47.0±0.3 ab | 3.0 |

Note: CF: conventional fertilization; RBIT: reduced basal and increased topdressing fertilizer rate; RBITS: RBIT combined with straw incorporation. MWD: mean weight diameter. Different lowercase letters represent significant differences (*P* ≤ 0.05) among treatments. Data: mean±standard error (SE).

**Table S2 Effect of fertilization management on photosynthesis parameters of sword leaf at rice heading stage (2017).**

| Treatment | Photosynthesis rate | Stomatal conductance | Transpiration rate |
| --- | --- | --- | --- |
| CF | 19.5±1.1 b | 0.9±0.07 b | 9.1±0.5 a |
| BRTI | 22.8±1.0 a | 1.2±0.07 a | 10.0±0.3 a |
| BRTS | 23.3±0.7 a | 1.1±0.05 a | 9.2±0.3 a |

Note: CF: conventional fertilization; RBIT: reduced basal and increased topdressing fertilizer rate; RBITS: RBIT combined with straw incorporation. MWD: mean weight diameter. Different lowercase letters represent significant differences (*P* ≤ 0.05) among treatments. Data: mean±standard error (SE).
